# Generation of functional salivary gland tissue from human submandibular gland stem/progenitor cells

**DOI:** 10.1186/s13287-020-01628-4

**Published:** 2020-03-20

**Authors:** Yi Sui, Siqi Zhang, Yongliang Li, Xin Zhang, Waner Hu, Yanrui Feng, Jingwei Xiong, Yuanyuan Zhang, Shicheng Wei

**Affiliations:** 1 Department of Oral and Maxillofacial Surgery and Central Laboratory, School and Hospital of Stomatology, Peking University, No. 22 Zhong-Guan-Cun South Road, Hai-Dian District, Beijing, 100081 China; 2 Laboratory of Biomaterials and Regenerative Medicine, Academy for Advanced Interdisciplinary Studies, Peking University, Beijing, 100871 China; 3 Biomedical Pioneering Innovation Center, and State Key Laboratory of Protein and Plant Gene Research, Peking University, Beijing, China; 4 Institute of Molecular Medicine, and State Key Laboratory of Natural and Biomimetic Drugs, Peking University, Beijing, 100871 China; 5 Wake Forest Institute for Regenerative Medicine, 391 Technology Way, Winston-Salem, NC USA

**Keywords:** Human salivary gland stem cells, Organoids, Salivary gland regeneration, Xerostomia, FGF10, Mouse embryonic salivary gland mesenchyme

## Abstract

**Background:**

Organ replacement regenerative therapy based on human adult stem cells may be effective for salivary gland hypofunction. However, the generated tissues are immature because the signaling factors that induce the differentiation of human salivary gland stem cells into salivary glands are unknown.

**Methods:**

Isolated human submandibular gland stem/progenitor cells (hSMGepiS/PCs) were characterized and three-dimensionally (3D) cultured to generate organoids and further induced by fibroblast growth factor 10 (FGF10) in vitro. The induced spheres alone or in combination with embryonic day 12.5 (E12.5) mouse salivary gland mesenchyme were transplanted into the renal capsules of nude mice to assess their development in vivo. Immunofluorescence, quantitative reverse transcriptase-polymerase chain reaction, calcium release analysis, western blotting, hematoxylin–eosin staining, Alcian blue–periodic acid-Schiff staining, and Masson’s trichrome staining were performed to assess the structure and function of generated tissues in vitro and in vivo.

**Results:**

The isolated hSMGepiS/PCs could be long-term cultured with a stable genome. The organoids treated with FGF10 [FGF10 (+) group] exhibited higher expression of salivary gland–specific markers; showed spatial arrangement of AQP5^+^, K19^+^, and SMA^+^ cells; and were more sensitive to the stimulation by neurotransmitters than untreated organoids [FGF10 (−) group]. After heterotopic transplantation, the induced cell spheres combined with mouse embryonic salivary gland mesenchyme showed characteristics of mature salivary glands, including a natural morphology and saliva secretion.

**Conclusion:**

FGF10 promoted the development of the hSMGepiS/PC-derived salivary gland organoids by the expression of differentiation markers, structure formation, and response to neurotransmitters in vitro. Moreover, the hSMGepiS/PCs responded to the niche in mouse embryonic mesenchyme and further differentiated into salivary gland tissues with mature characteristics. Our study provides a foundation for the regenerative therapy of salivary gland diseases.

## Background

Salivary glands are exocrine glands composed of acini, a duct system, myoepithelial cells, and interstitium. There are three major salivary glands—the parotid, submandibular, and sublingual glands—and minor salivary glands. They produce an average of 1–1.5 L of saliva daily, which is essential to the health and function of the gastrointestinal tract and oral cavity, including digestion of starch, normal speech, taste, mastication, swallowing, and the maintenance of teeth through its digestion antimicrobial, cleansing, lubricating, and buffering functions [1, 2]. Xerostomia, a subjective sensation of salivary gland dysfunction and resultant symptoms, can be caused by radiotherapy for head-and-neck cancer, surgical injury, Sjögren’s syndrome, genetic anomalies, or aging [3, 4]. Xerostomia greatly reduces the quality of life and general health status of patients due to various complications such as mastication and swallowing difficulties, hampered speech, deadened taste, oral infection, and caries [5, 6]. The available therapies for xerostomia—including artificial saliva substitutes, sialogogues, and systemic parasympathomimetic drugs—are palliative because their effect is temporary and salivary gland function is not restored [1, 7]. Stem cell-based regenerative medicine holds the promise for treating salivary gland hypofunction fundamentally.

Organoids are three-dimensional (3D) structures grown from pluripotent stem cells or adult stem cells and exhibit spatially arranged organ-specific cells in a manner similar to that in vivo and can be applied to new-generation regenerative therapy, development and disease modeling, and drug testing [8, 9]. Step-wise induction can cause mouse embryonic stem cells to develop into an early salivary gland by organoid technology and mature further after orthotopic transplantation [10], demonstrating the feasibility of regeneration of functional salivary glands for organ replacement therapy. However, concern over the carcinogenicity and tumorigenicity of mouse embryonic stem cells and the heterogeneity of animal-derived cells has hampered their application.

Human adult stem cells are homologous and low risk and have been used as an alternative to generate structure-specific and functional organoids of various organs, such as the intestine [11], pancreas [12], stomach, brain, liver, and lung [8]. In the human salivary gland, stem cells reside in the ductal region, as indicated by the co-localization of stem cell markers [13]. Although the universal markers for human salivary gland epithelial stem/progenitor cells are not available, these cells can be induced to differentiate into ductal cells, acinar cells, and even hepatocytes in vitro, showing their differentiation potential, and c-kit, Krt5 (K5), K14, CD49f, CD90, and CD44 were used as the stem/progenitor cell markers in these reports [14–17]. Regarding 3D culture of human salivary gland stem cells, Pringle and colleagues mixed Matrigel and collagen to drive differentiation of cultured spheres into branching and lobular structures [18]. Also, Srinivasan and co-workers used hyaluronate hydrogel to culture primary salivary gland stem/progenitor cells and drive its acinar-like differentiation [16]. Organogenesis is regulated by complex signaling communications among epithelium, mesenchyme, nerves, and vessels. However, the signals that define the fate of human submandibular gland epithelial stem/progenitor cells (hSMGepiS/PCs) are unclear and the hSMGepiS/PC-derived spheres lack a clear structure. Fibroblast growth factor 10 (FGF10) is implicated in mesenchymal signaling to the epithelium and is essential for mouse embryonic submandibular gland morphogenesis [19–21]. In humans, FGF10 mutation leads to aplasia of the lacrimal and salivary glands, and lacrimo-auriculo-dento-digital syndrome. Moreover, the mouse embryonic salivary gland mesenchyme induces reassembled embryonic epithelial cells and mouse embryonic stem cell–derived epithelium into functional submandibular glands [10, 22]. Whether human-derived adult epithelial stem cells respond to the mouse embryonic mesenchyme and their developmental level are important issues for the further chemically defined induction as bulk mechanism researches are from animal developmental models.

Here, we demonstrated that differentiation of hSMGepiS/PCs could be induced by FGF10. The cells self-organized into salivary gland organoids that exhibited increased expression of differentiation markers, a regular structure, and an elevated response to neurotransmitters in vitro. To further explore the development of hSMGepiS/PC-derived salivary gland tissues, the mouse embryonic salivary gland mesenchyme was introduced, and a structurally mature and functional salivary gland was generated in vivo. Therefore, signals from the mouse embryonic mesenchyme were applicative for the development of hSMGepiS/PCs. This study provides a foundation for the development of hSMGepiS/PC-based salivary gland regenerative therapy.

## Methods

### Salivary gland tissue

Human submandibular gland (hSMG) tissues were obtained from three patients (one male and two females, 20–40 years old) with ameloblastoma, in whom the affected part of the submandibular salivary gland had to be removed to facilitate reconstruction of mandibular defects. The patients provided informed consent, and the biomedical ethics committee of Peking University in China approved the studies. None of the patients was diagnosed with a salivary gland disease or systemic disease, and the ameloblastoma did not involve the salivary gland.

### Isolation and culture of epithelial stem cells and mesenchymal cells from hSMGs

hSMG tissues were collected after surgery and stored in Dulbecco’s modified Eagle’s medium (DMEM; HyClone) after surgery. To culture primary cells from hSMGs, the tissues were transferred to a biosafety cabinet, cut into small pieces, and placed at the bottom of culture bottles (Corning). After incubation at 37 °C in 5% CO_2_ for 4 h, epithelial cells were cultured in keratinocyte medium (KM; Sciencell) with supplements (KM-S) containing 5 μg/mL bovine serum albumin (BSA; Sigma-Aldrich), 5 ng/mL fibroblast growth factor-2 (FGF2; Sigma-Aldrich), 1 ng/mL epidermal growth factor (EGF; Sigma-Aldrich), 5 μg/mL insulin (Sigma-Aldrich), 5 μg/mL transferrin (Sigma-Aldrich), and 0.5 μg/mL hydrocortisone (Sigma-Aldrich); mesenchymal cells were cultured in DMEM-S, DMEM containing 10% fetal bovine serum (FBS; Gibco) and 1% penicillin/streptomycin (Invitrogen). The medium was changed every 3 days. Primary cells were digested with 0.25% trypsin (Invitrogen), harvested by centrifugation, resuspended in medium, and reseeded in a cell-culture dish as passage 1 (P1). The digestion time was 4 min for epithelial cells and 1.5 min for mesenchymal cells. Cells at 80–90% confluence were passaged at a 1:3 split ratio, and the medium was changed every 2–3 days.

### Cell self-renewal, proliferation, and viability assays

Sphere-forming assays were used to assay the self-renewal capacity of single cells in 3D culture. Cells were enzymatically dispersed into single cells, and 500 cells were mixed with 20 μL of Matrigel and 20 μL of medium, dropped into 96-well plates, and overlaid with 200 μL of medium. Images were obtained daily, and spheres > 40 μm were counted at days 5–7.

A Cell Counting Kit-8 (CCK-8) (Dojindo) was used to assay cell proliferation and viability. Cells were seeded at a density of 2 × 10^4^ per well in a 24-well plate and incubated for 7 days. Cells at passages 3, 6, 9, and 12 were washed twice in phosphate-buffered saline (PBS), and 500 μL of medium and 50 μL of CCK8 solution were added, followed by incubation at 37 °C in 5% CO_2_ for 2 h. The absorbance at 450 nm was read daily for 7 days. Each sample had three replicate wells. The doubling time was estimated using Eq. (1), where *T*_*d*_ denotes doubling time, *N*0 denotes absorbance during the logarithmic phase, *Nt* denotes absorbance after *N*0 during the logarithmic phase, and *∆t* denotes the time of *Nt* minus the time of *N*0.

1 $$ {T}_d=\varDelta t\times \mathit{\log}2\div \left( logNt- logN0\right) $$

### DNA-seq analysis

DNA sequencing (DNA-seq) was used to assess the genomic stability of long-term cultured hSMGepiS/PCs. Whole genomic DNA of hSMGepiS/PCs at P1, P8, and P16 was extracted using the Blood & Cell Culture DNA Midi Kit (Qiagen) and fragmented to 300 bp by Focused-ultrasonicators (Covaris). DNA library was prepared using the NEBNext Ultra DNA Prep Kit and sequenced on the Illumina Hiseq-PE150 platform. And genome copy number analysis was performed as reported previously [23].

### In vitro formation and culture of organoids

To form organoids, hSMGepiS/PCs at passages 3–8 were digested with 0.25% trypsin, collected by centrifugation, counted, and resuspended in KM-S to 5 × 10^7^/mL. Matrigel (cat# 356234, Corning) was mixed with medium (Matrigel: medium = 1:1) on ice, plated into 96-well ultra-low adherence plates (Corning) at 30 μL/well, and solidified for 30 min at 37 °C. Next, 0.1–0.2 μL of cell suspension was injected into the Matrigel drops using a 0.1–10-μL pipette tip, and 200 μL of medium was added. After 1 day, fibroblast growth factor 10 (FGF10; Sigma-Aldrich) was added and the medium was changed daily. The organoids were cultured for 14 days at 37 °C in 5% CO_2._

### RNA extraction, cDNA synthesis, and quantitative reverse transcriptase-polymerase chain reaction

Total RNA was extracted using TRIzol reagent (Invitrogen) according to the manufacturer’s instructions and reverse transcribed into cDNA using a RevertAid™ First-Strand cDNA Synthesis Kit (Thermo) with random hexamers and RNase according to the manufacturer’s protocol after determination of quality and concentration.

Polymerase chain reaction (PCR) amplification was performed using specific primers (see Additional file 1: Table S1). The reaction conditions were 2 min at 95 °C, followed by 25 cycles of 95 °C for 40 s, 55 °C for 40 s, and 72 °C for 14 s, and 72 °C for 5 min.

Quantitative reverse transcriptase-polymerase chain reaction (qRT-PCR) was performed according to the manufacturer’s protocol. The cycling conditions were 95 °C for 10 min, followed by 40 cycles of 95 °C for 5 s and 60 °C for 30 s. The comparative cycle threshold (Ct) method was used to calculate expression levels, which were normalized to that of 28S RNA.

### Calcium release analysis

The Fluo-4 Calcium Imaging Kit (Thermo) was used to assay the intercellular calcium release of organoids. Organoids were incubated with Fluo-4, AM for 1 h at 37 °C and then washed twice with DPBS. One hundred micromolar Carbachol (CCh) was added to stimulate calcium influx, and the change in signal was captured by live-cell imaging and confocal microscopy (Nikon).

### Hematoxylin and eosin and Masson’s trichrome staining

Organoids and salivary gland tissue were fixed in 4% (w/v) paraformaldehyde overnight at 4 °C and dehydrated in 10%, 20%, and 30% sucrose overnight at 4 °C. Next, the samples were cryosectioned at a thickness of 5–6 μm and embedded in optimum cutting temperature compound. Random sections were stained with hematoxylin and eosin (HE) staining according to standard protocols. For Masson’s trichrome staining, tissue sections were incubated in Ehrlich’s hematoxylin for 5 min and rinsed in tap water. Sections were then incubated for 5 min in a 2:1 mixture of 1% (w/v) acid fuchsin (Sigma-Aldrich) in 1% (v/v) glacial acetic acid with 1% Ponceau xylidine (Sigma-Aldrich) in 3% glacial acetic acid. After washing in deionized water, sections were incubated for 1 min in 1% Aniline Blue (Sigma-Aldrich) in 3% glacial acetic acid, washed in deionized water, and finally incubated for 5 min in 1% (w/v) molybdenum phosphoric acid and again washed.

### Immunofluorescence and confocal microscopy

Cells were fixed in 4% paraformaldehyde for 30 min at room temperature and washed several times in PBS. Cells or tissue sections were permeabilized for 10 min in 0.1% Triton X-100 (Sigma-Aldrich) and blocked in 5% BSA for 2 h at room temperature. Primary antibodies against K5 (cat# ab128190, Abcam), K19 (cat# ab52625, Abcam), CD49f (cat# ab18155, Abcam), human-specific E-cadherin (cat# ab40772, Abcam), AQP5 (cat# ab78486, Abcam), α-SMA (cat# ab7817, Abcam), ZO1(cat# ab59720, Abcam), CD73 (cat# ab81720, Abcam), and CD90 (cat# ab133350, Abcam) in a mixture of equal volumes of 0.1% Triton X-100 and 5% BSA were incubated at 4 °C overnight. Following three washes for 15 min each in PBS, the samples were incubated for 2 h at room temperature with one of the following secondary antibodies (ZSGB-BIO): fluorescein-conjugated goat anti-mouse IgG (488 nm) and Rhodamine-conjugated goat anti-rabbit IgG (543 nm) at 1:200 dilution. Phalloidin (Sigma-Aldrich) was used to stain F-actin and 4′,6-diamidino-2-phenylindole (DAPI; Sigma-Aldrich) for nuclei.

### Alcian blue–periodic acid-Schiff staining

Alcian blue–periodic acid-Schiff (AB-PAS) staining was used to assess the secretion of saliva from organoids and regenerated salivary glands. Sections were stained with AB and PAS reagent for 30 min. Next, the samples were rinsed in tap water for 10 min, oxidized in periodic acid (5 g/L) for 5 min, rinsed in lukewarm tap water for 10 min, and stained with Coleman’s Schiff reagent for 10 min.

### Western blot analysis

Total protein was extracted using radioimmunoprecipitation assay buffer lysis buffer (Solarbio) containing a protease inhibitor (cat# P8340, Sigma-Aldrich), and the protein concentration was determined by bicinchoninic acid assay (Thermo). Equal amounts of denatured total protein were subjected to sodium dodecyl sulfate-polyacrylamide gel electrophoresis at 100 V. Following transfer onto polyvinylidene difluoride membranes, nonspecific binding sites were blocked, and the blots were incubated with monoclonal antibodies against K5, K19, CD49f, CD73, CD90, and Ascl3 (cat# ab166820, Abcam) overnight. Next, the secondary antibody was added, and bands were developed by enhanced chemiluminescence according to the manufacturer’s instructions. To quantify band intensity, densitometry with correction for protein loading was performed using Image Gauge software (LAS-1000plus, Fujifilm, Tokyo, Japan).

### Transplantation

hSMGepiS/PC spheres treated with FGF10 were harvested from Matrigel at day 3. The spheres alone or with mouse E12.5 submandibular gland mesenchyme were transplanted into the renal capsules of nude mice and raised for 30 days.

### Statistical analysis

Statistical analysis was performed by *t* test for two independent samples [FGF10 (+) group and FGF10 (−) group] and by one-way analysis of variance for more than two samples using SPSS. A *P* value of < 0.05 was indicative of statistical significance. Results are shown as means ± standard deviation (SD).

## Results

### Isolation, identification, and characterization of hSMGepiS/PCs

**Fig. 1:**
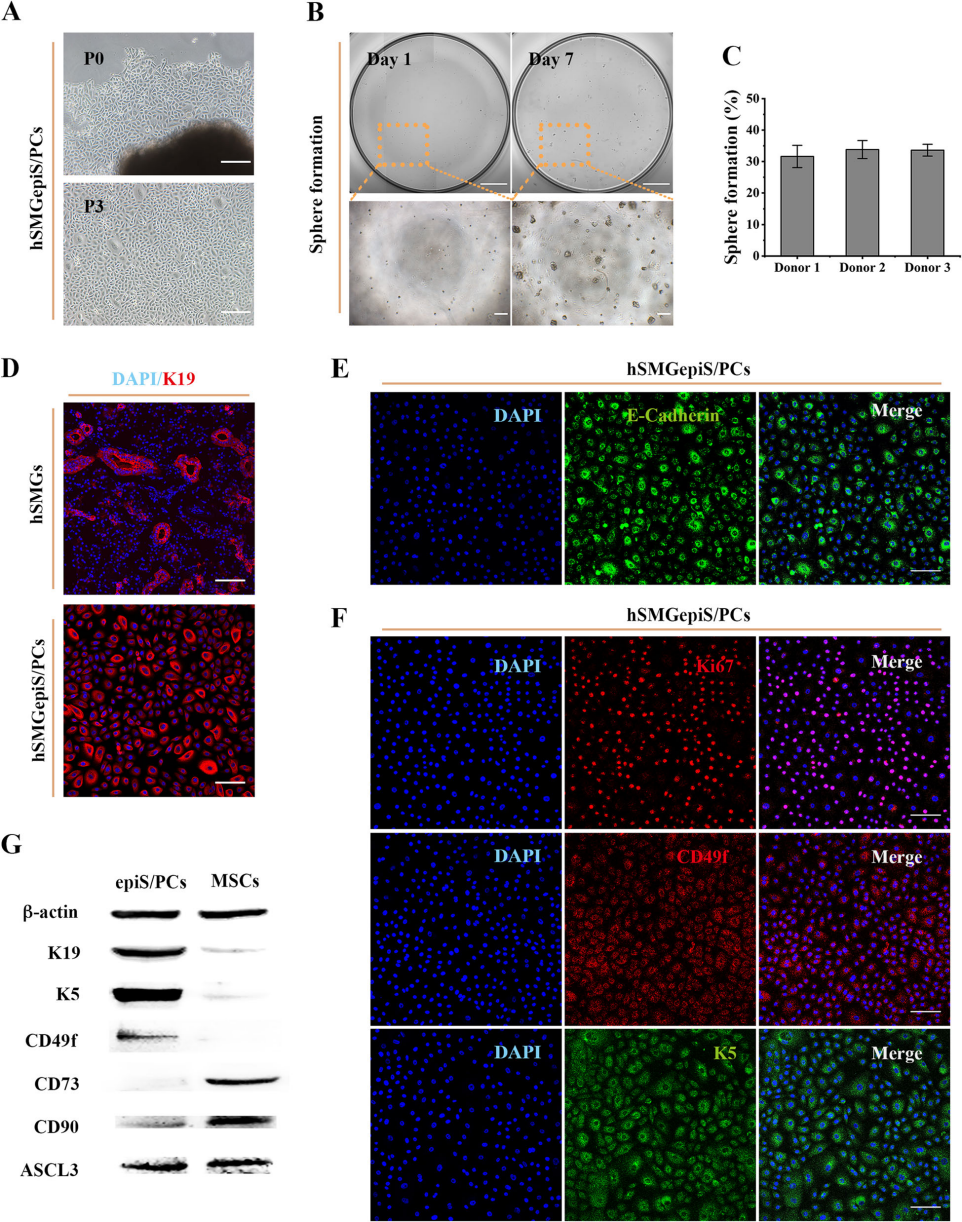
Isolation, identification, and characterization of hSMGepiS/PCs. **a** Images of primary cells isolated and passaged in 2D culture. Scale bar = 200 μm. **b** Representative images of single cells 3D-cultured and spheres formed. Scale bar = 1 mm (above) and 200 μm (below). **c** Quantification of sphere formation of hSMGepiS/PCs from different donors at day 7 after 3D culture. Error bars, SD, *n* = 6. **d** Immunofluorescence of K19 in cells and hSMGs to locate the isolated cells. Scale bar = 100 μm. **e** Positive expression of an epithelial marker, E-cadherin, in isolated cells. Scale bar = 100 μm. **f** Immunofluorescence showing that isolated cells express the proliferation marker, Ki67, and the epithelial stem/progenitor cell markers, K5 and CD49f. Scale bar = 100 μm. **g** Protein levels of the indicated markers in hSMGepiS/PCs and hSMGMCs by western blotting

KM medium with supplements was used to isolate epithelial cells from pieces of hSMGs, and cobblestone-shaped cells migrated after 6–8 days. These cells could be passaged (Fig. 1a) and yielded obvious clones at the single-cell level in 3D culture at day 7 (Fig. 1b), suggesting a self-renewal capacity. The sphere-formation rate reached 30.93 ± 5.98% and did not differ according to donor (Fig. 1c). As indicated by immunofluorescence, all the cultured cells were positive for K19 (epithelial lineage-related duct-specific marker), and ductal cells were detected in hSMG tissue, indicating a ductal origin (Fig. 1d). Notably, a fraction of larger cells, which tended to be differentiated cells, showed higher K19 expression. Moreover, their positivity for E-cadherin (calcium-dependent cell adhesion protein of epithelial cells) showed the isolated cells to be epithelial cells (Fig. 1e). The expression of Ki67 (a proliferation marker), K5 and CD49f (epithelial stem/progenitor markers of hSMGepiS/PCs) [14, 16, 24], and Ascl3 (an adult stem/progenitor marker) [25, 26] was tested by immunofluorescence and western blotting to evaluate stemness. The isolated epithelial cells were positive for these markers (Fig. 1f, g). Fibroblast-like cells were isolated and cultured for up to 15 passages without obvious changes in morphology or doubling time, and were positive for CD73 and CD90 (mesenchymal stem cell markers) by immunofluorescence in DMEM-S, indicating them to be human submandibular gland mesenchymal cells (hSMGMCs) [27] (see Additional file 1: Figure S1). A protein analysis was performed to distinguish the epithelial cells from the mesenchymal cells. The isolated epithelial cells exhibited high K5, CD49f, and Ascl3 protein levels and a low level of CD90 protein, which was also reported as a progenitor marker in human salivary glands [13]. CD73 protein was not detected (Fig. 1g). By contrast, hSMGMCs expressed CD73, CD90, and Ascl3 proteins, but not K5 or CD49f (Fig. 1g). Therefore, the isolated cells showed epithelial stem/progenitor cell characteristics and were successfully separated from mesenchymal cells.

### Long-term culture of hSMGepiS/PCs

**Fig. 2:**
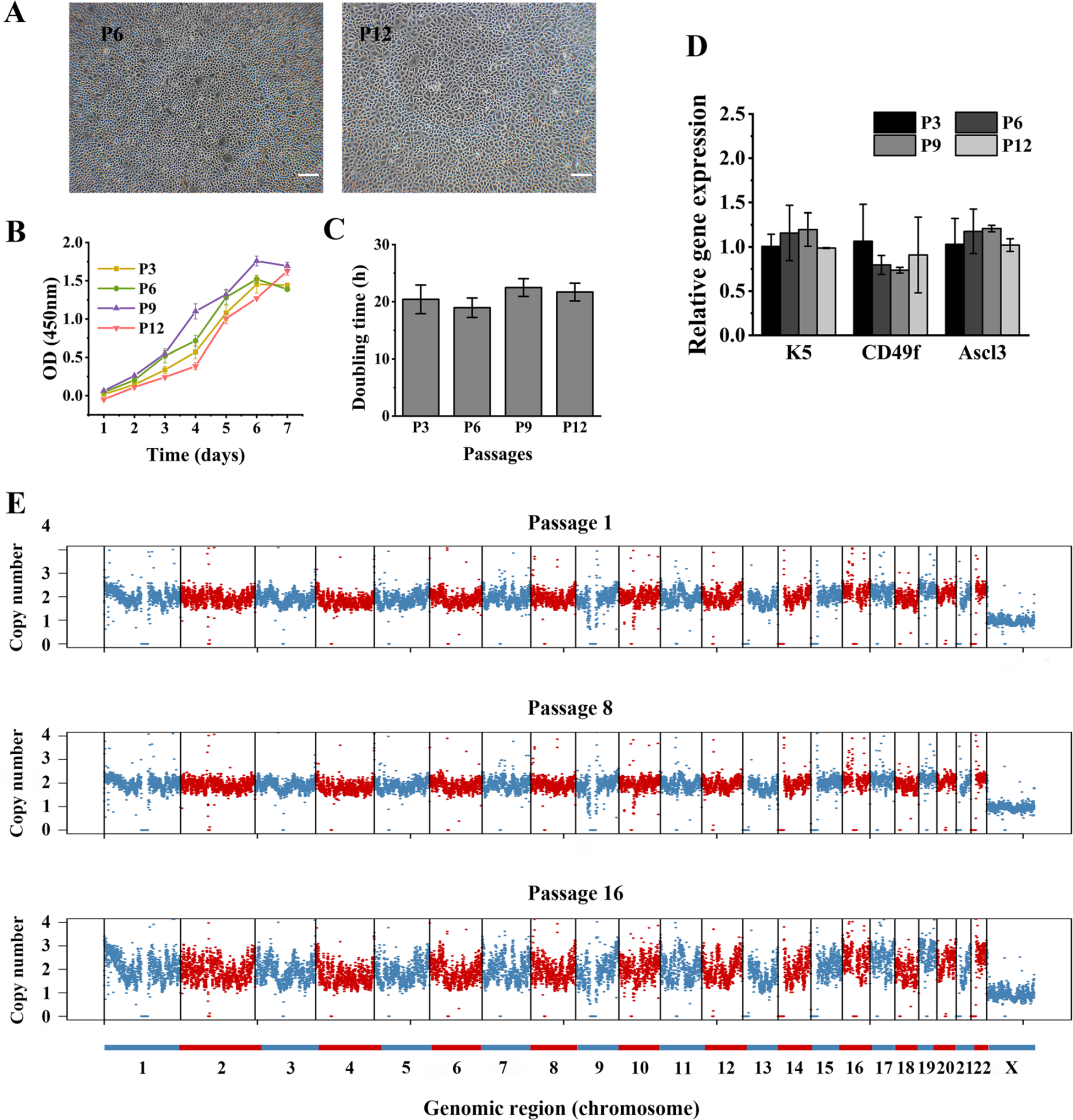
Long-term culture of hSMGepiS/PCs. **a** Images of long passaged hSMGepiS/PCs in 2D culture. Scale bar = 200 μm. **b** Growth curve and **c** doubling time of passaged hSMGepiS/PCs in 2D culture. Error bars, SD. Statistical analysis was performed using ANOVA, *F* = 1.266, *P* = 0.357, *n* = 3. **d** qRT-PCR showing that molecular markers of stem/progenitor cells were intact after long-term culture. Error bars, SD. ANOVA, *F* = 1.820 and *P* = 0.222 (K5), *F* = 1.183 and *P* = 0.383 (CD49f), *F* = 1.467 and *P* = 0.295 (Ascl3), *n* = 3. **e** Bulk DNA-seq results of hSMGepiS/PCs at passages 1, 8, and 16. Chromosomes are indicated by interlaced colors. Points are bins of 200 kb length

hSMGepiS/PCs were long passaged in vitro, during which they assumed the shape of a small cobblestone (Fig. 2a). The proliferation and expression levels of stem/progenitor markers of hSMGepiS/PCs with increasing passage number were investigated by CCK8 assay and qRT-PCR, respectively. The growth curves were sigmoidal (Fig. 2b), and the cell doubling times did not differ significantly at passages 3, 6, 9, and 12 (Fig. 2c). There was no significant difference in the expression of K5, CD49f, and Ascl3 according to the passage number (Fig. 2d), demonstrating that hSMGepiS/PCs could be maintained and expanded in vitro. Further, DNA-seq analysis was performed to evaluate the copy number variation of long-term cultured hSMGepiS/PCs. Although the bin counts of cells at P16 were dispersed, the trend was consistent irrespective of passage number, and they harbored two copies of chromosomes 1–22 and one copy of the X chromosome (Fig. 2e), indicating no copy number variation.

### Promotion of the development of the hSMGepiS/PC-derived salivary gland organoids by FGF10

**Fig. 3:**
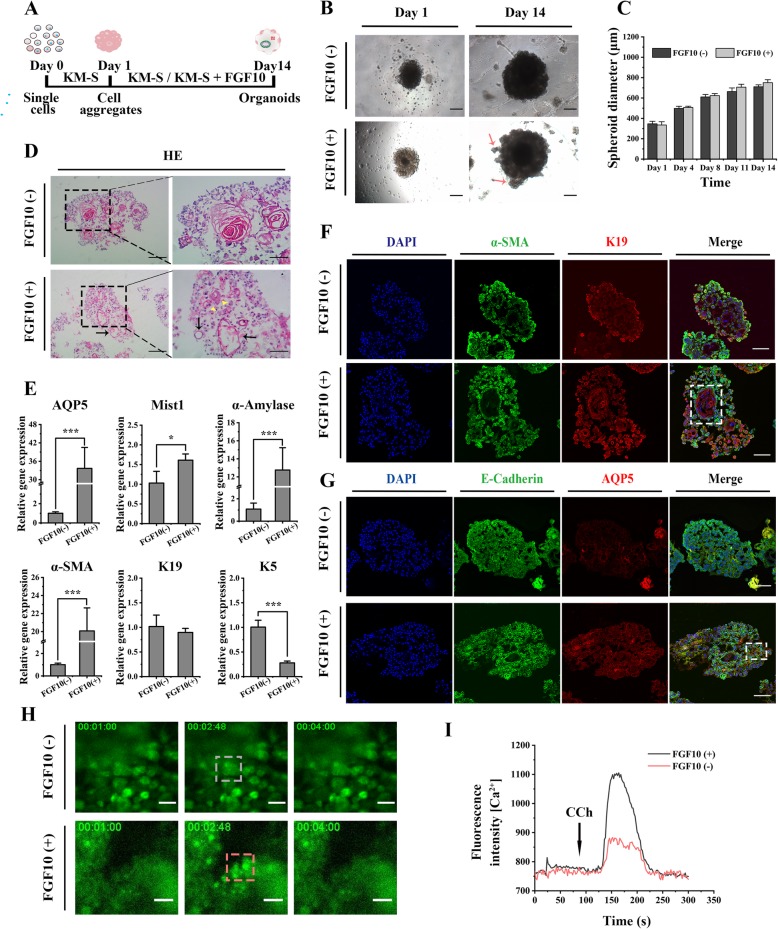
Development of the hSMGepiS/PC-derived salivary gland organoids with induction of FGF10 in vitro. **a** Schematic diagram of organoid formation and induction. **b** Bright-field image of an organoid with emerged buds (red arrows) with FGF10 induction at day 14. Scale bar = 200 μm. **c** Quantification of sphere size following 3D culture. Error bars, SD, *n* = 3. **d** HE staining of organoids at day 14 showing both acinar-like (yellow arrowheads) and ductal-like (black arrows) structures. Scale bar = 200 μm (left), 100 μm (right). **e** Increased expression of molecular markers of mature salivary glands and decreased expression of a progenitor marker with FGF10 induction by qRT-PCR. Error bars, SD, ***P < 0.01, *P < 0.05, *n* = 3. **f** Representative immunofluorescence images of the expression and location of K19 and α-SMA (white rectangle). Scale bar = 100 μm. **g** Immunofluorescence images of E-cadherin^+^ and AQP5^+^ cells in different organoids. White rectangle indicated located AQP5^+^ cells. Scale bar = 100 μm. **h** Immunofluorescence images of different organoids before and after the CCh stimulation at 80 s. Scale bar = 50 μm. **i** Fluorescence intensity of both FGF10-treated and FGF10-untreated organoids after CCh stimulation

To explore its effect on the development of organoids in vitro, FGF10 was added to KM-S medium to culture hSMGepiS/PC-derived spheres from days 1 to 14. KM-S medium was used as the control (Fig. 3a). Cell aggregates with an irregular surface formed 1 day after seeding into Matrigel and grew to 600–800 μm irrespective of the presence of FGF10 (Fig. 3b, c). Interestingly, buds were generated only by spheres induced by FGF10 after 14 days (Fig. 3b). Although cells in all spheres self-organized into morphologically acinar-like and ductal-like structures after 14 days in 3D culture, the lumens of those in the presence of FGF10 were more structured with regular edges (Fig. 3d).

The degree of differentiation of organoids was evaluated by qRT-PCR and immunofluorescence. The expression of AQP5 and Mist1 (acinar markers), α-amylase (functional marker), and α-SMA (salivary myoepithelial marker) was significantly increased, and the expression of a progenitor marker, K5, was decreased by FGF10 (Fig. 3e), demonstrating that FGF10 contributed to the differentiation of acinar and myoepithelial cells from hSMGeipS/PCs. The expression of K19 was unchanged. Additionally, immunofluorescence showed that the spheres treated with FGF10 consisted of K19^+^, α-SMA^+^, AQP5^+^, and E-cadherin^+^ cells, which were spatially distributed in different locations. K19^+^ cells formed ductal-like structures surrounded by α-SMA^+^ cells, and AQP5^+^ cells were localized at the acinar-like structures (distant from the ductal-like structures), in FGF10-treated spheres (Fig. 3f, g). Differently, both the expression of AQP5 and the spatial arrangement in untreated spheres were too weak to be visualized (Fig. 3f, g).

Saliva secretion by the salivary glands is stimulated by the peripheral nervous system [28]. We next evaluated the function of organoids by measuring CCh-induced calcium release. More cells responded to transient induction of CCh (Fig. 3h), and calcium release was higher in FGF10-treated than in untreated organoids (Fig. 3i). Therefore, FGF10 promoted the differentiation of hSMGepiS/PC-derived salivary gland organoids in terms of gene expression, morphogenesis, and response to neurotransmitters.

### In vivo generation of structural and functional salivary glands combined with mouse embryonic mesenchyme

**Fig. 4:**
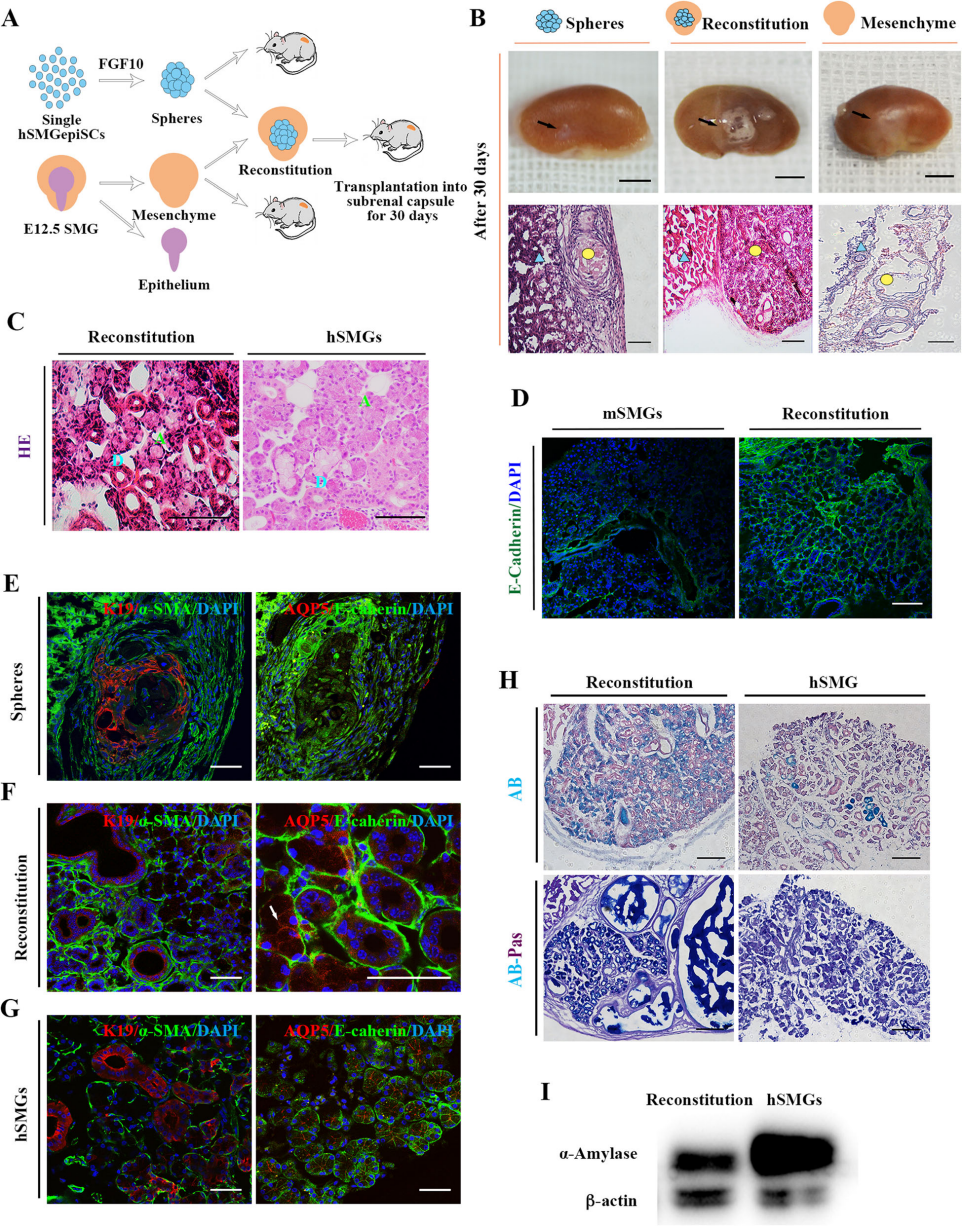
In vivo generation of salivary gland tissues from the hSMGepiS/PCs. **a** Schematic diagram of reconstitution and transplantation. **b** Representative photographs of mouse kidneys with renascent white tissue (black arrows) at day 30 after transplantation of hSMGepiS/PC spheres alone, E12.5 mesenchyme alone, and reconstitution of the above two tissues (top). Scale bar = 4 mm. Morphology of corresponding renascent tissue as visualized by HE staining (bottom). Blue triangle, kidney tissue; yellow circle, renascent tissue. Scale bar = 200 μm. **c** HE staining showing ductal and acinar structures in the reconstitution group (right) similar to natural human submandibular gland tissue (left). Blue D, ducts; green A, acini. Scale bar = 100 μm, *n* = 6. **d** Human-specific E-cadherin staining of a regenerated salivary gland in the reconstitution group (bottom). Mouse SMGs (mSMGs) were used as the negative control (top). Scale bar = 100 μm. **e**–**g** Immunofluorescence of the generated tissues in the hSMGepiS/PC spheres group (**e**) and the reconstitution group (**f**) for the salivary markers E-cadherin, AQP5 (write arrow), α-SMA, and K19, compared with human salivary glands (**g**). Scale bar = 50 μm. **h** AB-PAS staining showing comparable expression of glycoproteins in generated glands from the reconstitution group and hSMGs. Scale bar = 200 μm. **i** Western blot showing expression of amylase in the reconstitution-derived glands; hSMGs was used as the positive control

We next investigated whether the FGF10-induced spheres derived from hSMGepiS/PCs could further develop in vivo following transplantation into the mouse renal capsule for 30 days. Moreover, E12.5 mouse submandibular gland mesenchyme was added to test whether the human adult cell–derived spheres responded to the dynamic niche in mouse mesenchyme (reconstitution group). Mesenchyme tissues were transplanted alone as the control (Fig. 4a). E12.5 mouse submandibular gland epithelium was separated from the mesenchyme (Additional file 1: Figure S2). At 30 days, the generated tissues on the surface of the kidneys of the three groups were visually different. Protuberant white tissues with vacuoles were noticeable in the reconstitution group (Fig. 4b). Histological analysis showed that the FGF10-induced spheres did not spontaneously generate salivary gland–like tissue in vivo (Fig. 4b). Surprisingly, HE staining showed that the generated tissues in the reconstitution group were morphologically similar to the natural human submandibular glands, which have ductal and acinar structures (Fig. 4b, c). Transplantation of E12.5 mouse submandibular gland mesenchyme alone was performed to test whether there was residual embryonic epithelium in the isolated mesenchyme. The produced tissues contained only an interstitial structure with collagenous fibers (Fig. 4b), which was confirmed by Masson’s trichrome staining (Additional file 1: Figure S3). The expression of human-specific E-cadherin, which was negative in mouse SMG (Fig. 4d), indicated that the generated epithelial tissues in the reconstitution group were derived from human cells not from mouse embryonic mesenchyme.

The expression of salivary gland–specific markers was further tested. The spatial arrangement of α-SMA^+^ cells and K19^+^ cells also appeared in transplanted FGF10-treated spheres (Fig. 4e). However, the expression of AQP5 was weak and not a location-specific expression (Fig. 4e), due to the absence of continuous stimulation by FGF10 in vivo. However, when combined with E12.5 mouse submandibular gland mesenchyme, the generated glands were positive for AQP5 in the acinus, K19^+^ cells were selectively localized at the ductal structures, and basal localization of α-SMA^+^ cells around acini and ducts was detected (Fig. 4f), as observed in the human submandibular glands (Fig. 4g). Of note, the sections also exhibited a multilevel ductal structure (Fig. 4b, c, f).

Finally, we tested proteins and glycoproteins by AB-PAS staining and amylase secretion by western blotting, to evaluate the function of the regenerated tissue from reconstituted tissues. In the regenerated glands, much more secreta were stained than in the natural glands, and a retention pond formed (Fig. 4h). In addition, western blotting showed that the regenerated salivary glands secreted a large quantity of amylase (Fig. 4i). Therefore, all the results indicated that the hSMGepiS/PC-derived spheres responded to the mouse embryonic salivary gland mesenchyme and develop into a salivary gland of the correct structure with independent secretory function in vivo.

## Discussion

In this study, we generated further-differentiated human salivary gland organoids from hSMGepiS/PCs with induction of FGF10 in vitro. Also, a near-physiological salivary gland with correct structure and secretory function was regenerated in vivo from hSMGepiS/PCs plus mouse E12.5 salivary gland mesenchyme. Therefore, the dynamic niche in the developing mouse embryonic salivary gland facilitates the organogenesis of salivary glands from hSMGepiS/PCs.

Three-dimensional culture enables recapitulation of the in vivo cell morphology and cell–cell and cell–matrix interactions. Moreover, human adult stem cells from various organs can develop into organoids, which can be used for drug discovery, modeling, and next-generation regenerative medicine [8, 29]. Many efforts have been exerted for the 3D culture of human salivary gland stem cells and present the expression of acinar markers and ductal markers [16, 18]. The defined factors involved are unclear, and so further study is needed; organogenesis is strictly regulated by transcriptional networks. FGF10/FGFR2b signaling is essential in epithelial branching and histodifferentiation during mouse embryonic salivary gland morphogenesis [30]. FGF10 is also involved in the dynamic relationship of epithelium and mesenchyme at initial stage; later, the FGF10 signal is from the mesenchyme to the epithelial in mouse [31]. We evaluated the role of FGF10 in the development of hSMGepiS/PC-derived organoids. FGF10 induced the formation of buds and regular ductal-like structures, which contained location-specific K19^+^ cells and α-SMA^+^ cells, at day 14. The increased expression of acinar and myoepithelial markers demonstrated that FGF10 contributed to the differentiation of acinar and myoepithelial cells from hSMGeipS/PCs. Similar expression of K19 was speculated as the result was from bulk analysis and its innate high expression in hSMGepiS/PCs might obscure the trend. Moreover, the increased number and intensity of neurotransmitter-responding cells indicated FGF10 promoted the function of organoids. The establishment of mature human salivary gland organoids will likely allow regeneration of hypofunctional salivary glands. However, the salivary gland–like tissues lacked a mature structure because of nutrition limitations in vitro and the absence of further induction. Therefore, the FGF10-treated hSMGeipS/PCs were next transplanted into the renal capsule of nude mice. However, even the vessels exist, promotion of the maturity of hSMGeipS/PC-derived spheres in vivo requires other factors.

During natural submandibular gland development, after the invagination of the oral epithelium into mesenchyme at E11, the mesenchyme begins to condense and an early initial epithelial bud generates at E12. A branching structure is then formed, and terminal differentiation and functional maturation ensue [32]. Fetal development of epithelial stem/progenitor cells requires bidirectional signaling networks, including both secreted factors and physical interactions, with mesenchyme [32, 33]. E12.5 mouse SMG mesenchyme was introduced to induce the transplanted hSMGepiS/PC-derived spheres under the renal capsules of nude mice. Interestingly, near-physiological salivary gland tissues were formed, which exhibited a morphology and phenotype similar to those of human salivary glands, with a strong secretory function. The greater quantities of glycoproteins in regenerated glands compared to natural salivary glands were speculated to be due to the enrichment of secreta without excretion. Our findings demonstrate the strong regenerative potential of hSMGepiS/PCs. However, although the epithelial cells are human-derived, the animal-derived mesenchyme that induces their further development cannot be applied clinically, and its effect may involve the coordination of mesenchyme, nerves, and blood vessels. Further research is needed to define other proteins or macromolecules and their interactions to formulate the optimal in vitro differentiation strategy. The successful combination of human salivary gland cells with mouse embryonic mesenchyme provides a proof for the concept that the development of hSMGepiS/PC-derived organoids corresponds to that of mouse embryonic salivary glands. As in development of the mouse salivary gland, coordination of FGF and Wnt signaling regulates epithelial branching and salivary gland lumen formation, and vasoactive intestinal peptides induce the formation of a contiguous lumen [34, 35]. Moreover, not only nuclear factor IB is involved in, but also SOX2 regulates acinar cell development in the mouse salivary gland [36, 37]. The abundant mechanisms could be applied to the maturity of salivary gland organoids from human salivary gland stem cells in vitro and further confirmed in such human-resource modeling.

## Conclusion

FGF10 promoted the development of hSMGepiS/PC-derived salivary gland organoids in terms of morphogenesis and the response to neurotransmitters. Also, a salivary gland with mature characteristics was generated from hSMGepiS/PCs combined with mouse embryonic salivary gland mesenchyme, not only confirming the regenerative potential of hSMGepiS/PCs but also suggesting that hSMGepiS/PCs respond to the mouse embryonic mesenchyme niche and further differentiate. Therefore, our work will facilitate the regeneration of mature human salivary glands.

## Supplementary information

**Additional file 1: Figure S1.** Long-term culture and characterization of hSMGMCs. **a** Long-term culture of hSMGMCs in DMEM-S; scale bar = 200 μm. **b** Growth curve and (**c**) doubling time of passaged mesenchymal cells. Error bars, SD. Statistical analysis was performed by ANOVA, F = 1.160, P = 0.383, n = 3. **d** Immunofluorescence of CD73 and CD90 in hSMGMCs at passage 6 in 2D culture. Scale bar = 100 μm. **Figure S2.** Bright-field image of isolated E12.5 mouse SMG (left) and separation of the epithelium and mesenchyme (medium and right). Scale bar = 200 μm. **Figure S3.** Characterization of white area derived from E12.5 mouse submandibular gland mesenchyme after transplantation. Masson’s trichrome staining shows abundant collagenous fibers in renascent tissue of E12.5 mesenchyme (green circle), likely the interstitium of salivary glands (yellow triangle). Pink square, parenchyma of SGs; blue arrow, kidney. Scale bar = 200 μm. **Table S1.** Primer sequences used for PCR in this study.

## Data Availability

All data are included in this article and its supplementary information files.

## Abbreviations

3D: Three dimensional
AB-PAS: Alcian blue–periodic acid-Schiff
AQP5: Aquaporin-5
Ascl3: Achaete-scute homolog 3
BSA: Bovine serum albumin
CCh: Carbachol
DMEM-S: Dulbecco’s modified Eagle’s medium with supplements
DNA-seq: DNA sequencing
E12.5: Embryonic day 12.5 days
EGF: Epidermal growth factor
FBS: Fetal bovine serum
FGF10: Fibroblast growth factor 10
FGF2: Fibroblast growth factor 2
FGFR2b: Fibroblast growth factor receptor 2b
HE: Hematoxylin and eosin staining
hSMG: Human submandibular gland
hSMGepiS/PCs: Human submandibular gland epithelial stem/progenitor cells
hSMGMCs: Human submandibular gland mesenchymal cells
K19: Keratin 19
K5: Keratin 5
KM: Keratinocyte medium
KM-S: Keratinocyte medium with supplements
mSMG: Mouse submandibular gland
P: Passage
PCR: Polymerase chain reaction
qRT-PCR: Quantitative real-time polymerase chain reaction
SG: Salivary gland
α-AMY: α-Amylase
α-SMA: α-Smooth muscle actin
