# Supplementary material for: Generation of functional salivary gland tissue from human submandibular gland stem/progenitor cells
Source: Stem Cell Res Ther. 2020 Mar 20;11:127. doi: 10.1186/s13287-020-01628-4 (PMC7083056; doi:10.1186/s13287-020-01628-4)
Supplement: Supplementary file 1 — Additional file 1: Figure S1. Long-term culture and characterization of hSMGMCs. a Long-term culture of hSMGMCs in DMEM-S; scale bar = 200 μm. b Growth curve and (c) doubling time of passaged mesenchymal cells. Error bars, SD. Statistical analysis was performed by ANOVA, F = 1.160, P = 0.383, n = 3. d Immunofluorescence of CD73 and CD90 in hSMGMCs at passage 6 in 2D culture. Scale bar = 100 μm. Figure S2. Bright-field image of isolated E12.5 mouse SMG (left) and separation of the epithelium and mesenchyme (medium and right). Scale bar = 200 μm. Figure S3. Characterization of white area derived from E12.5 mouse submandibular gland mesenchyme after transplantation. Masson’s trichrome staining shows abundant collagenous fibers in renascent tissue of E12.5 mesenchyme (green circle), likely the interstitium of salivary glands (yellow triangle). Pink square, parenchyma of SGs; blue arrow, kidney. Scale bar = 200 μm. Table S1. Primer sequences used for PCR in this study. [file 13287_2020_1628_MOESM1_ESM.docx]

**Additional file 1:**


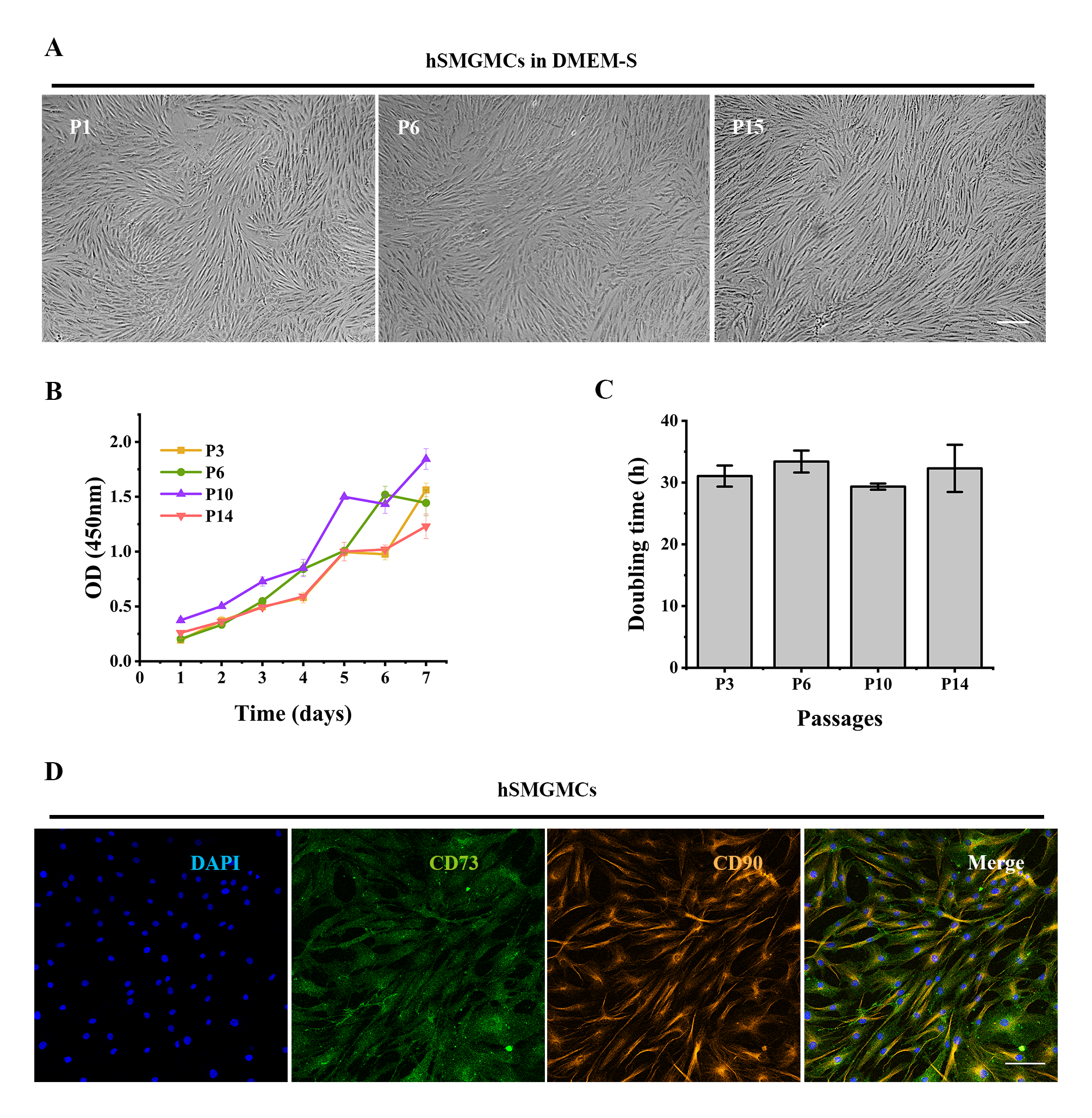


**Figure S1.** Long-term culture and characterization of hSMGMCs.

**a** Long-term culture of hSMGMCs in DMEM-S; scale bar = 200 μm.

**b** Growth curve and (**c**) doubling time of passaged mesenchymal cells. Error bars, SD. Statistical analysis was performed by ANOVA, F = 1.160, P = 0.383, n = 3.

**d** Immunofluorescence of CD73 and CD90 in hSMGMCs at passage 6 in 2D culture. Scale bar = 100 μm.

**
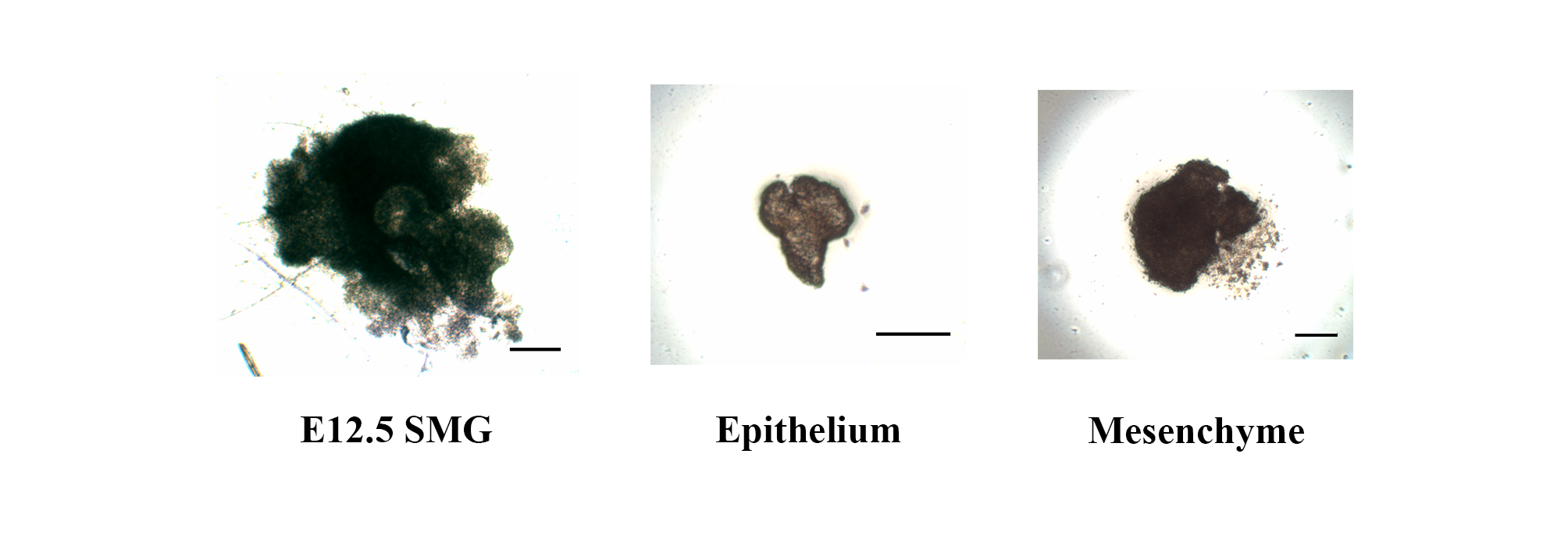
**

**Figure S2.** Bright-field image of isolated E12.5 mouse SMG (left) and separation of the epithelium and mesenchyme (medium and right). Scale bar = 200 μm.

**
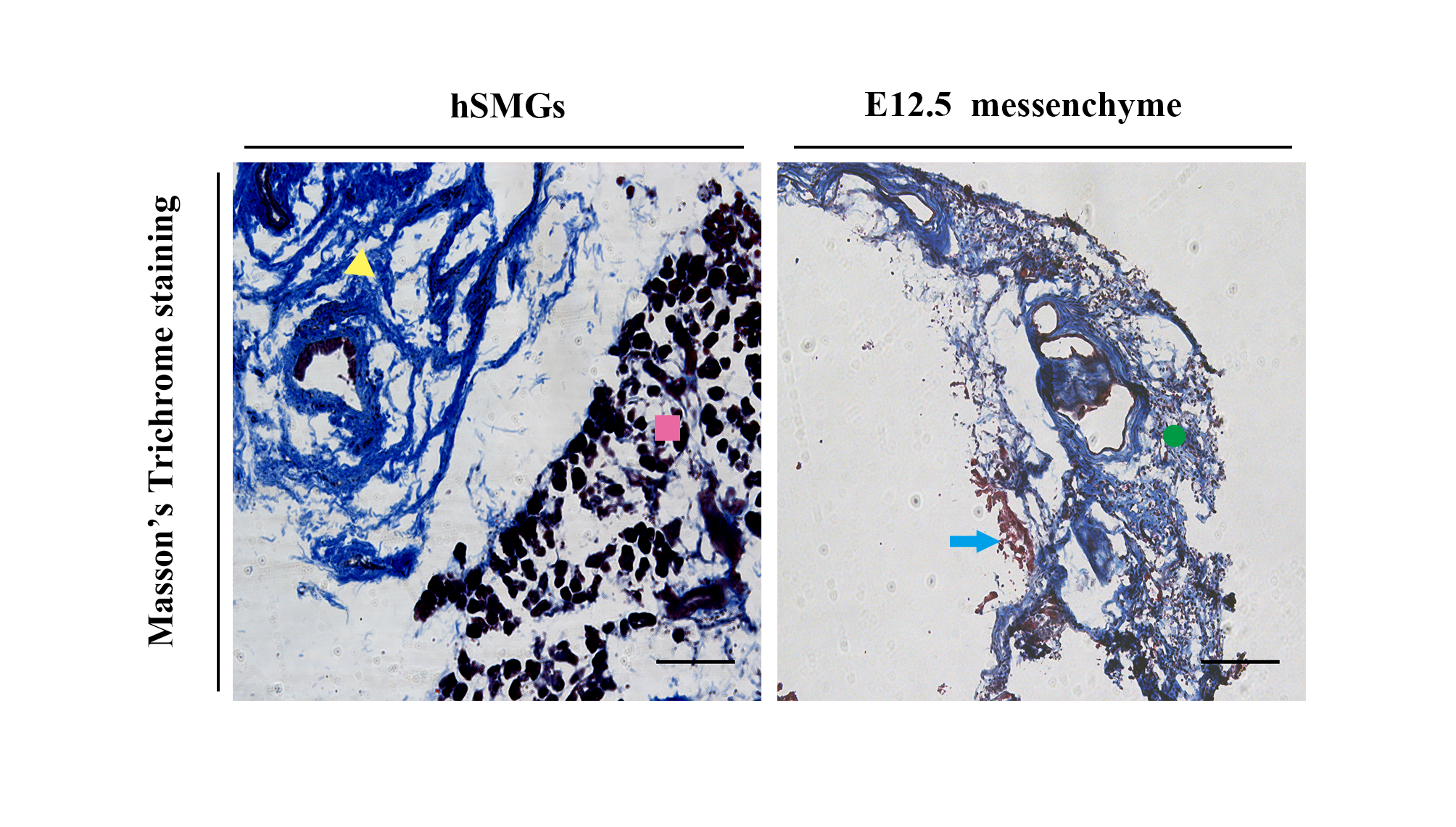
**

**Figure S3.** Characterization of white area derived from E12.5 mouse submandibular gland mesenchyme after transplantation.

Masson’s trichrome staining shows abundant collagenous fibers in renascent tissue of E12.5 mesenchyme (green circle), likely the interstitium of salivary glands (yellow triangle). Pink square, parenchyma of SGs; blue arrow, kidney. Scale bar = 200 μm.

**Table S1.** Primer sequences used for PCR in this study.

| Gene name | Forward primer | Reverse primer |
| --- | --- | --- |
| 28sr RNA | CCCAGTGCTCTGAATGTCAA | AGTGGGAATCTCGTTCATCC |
| K5 | CCGCTCACTGGGTTTTCTGG | GAGGAATGCAGACTCAGTGGA |
| K19 | TGAGGAGGAAATCAGTACGCT | CGACCTCCCGGTTCAATTCT |
| CD49f | AAGCGGCTGTTGCTCGTGGG | TTGCCCCCTGGACCTTGGCT |
| CD90 | CCTCCCTGCCTCCACCCACA | GCAGGGGCTGGCACTGATGG |
| CD105 | GCAATGAGGCGGTGGTCAAT | AGGAAGTGTGGGCTGAGGTA |
| Ascl3 | TGATCTGCCTGCCTCGGCCT | ACTCCTCCCCACCCCTCCCA |
| α-SMA | CCAGTGTGGAGCAGCCCAGC | CGGTGGACAATGGAAGGCCCG |
| α-amylase | AATTGATCTGGGTGGTGAGC | CTTATTTGGCGCCATCGATG |
| AQP5 | CCGCTCACTGGGTTTTCTGG | TTTGATGATGGCCACACGCT |

Abbreviations: K5: keratin 5; K19: keratin 19; Ascl3: achaete-scute homolog 3; α-SMA: α-smooth muscle actin; α-AMY: α-amylase; AQP5: aquaporin-5.
